# Extremely Low Frequency Radiation Enhances Soybean Chlorophyll Index and Nutrient Use Efficiency Under Suboptimal Conditions

**DOI:** 10.3390/plants15030495

**Published:** 2026-02-05

**Authors:** Fernanda Miotti, Rodrigo Lemos Lovato, Luzo Dantas Júnior, Adriana Sturion Lorenzi, Tiago Tezotto, Ricardo Ferraz de Oliveira

**Affiliations:** 1 Department of Biological Science, “Luiz de Queiroz” College of Agriculture (Esalq), University of São Paulo (USP), Piracicaba 13418-900, SP, Brazil; 2 Department of Soil Science, “Luiz de Queiroz” College of Agriculture (Esalq), University of São Paulo (USP), Piracicaba 13418-900, SP, Brazil; 3 Effatha Agro, Santo André 09030-320, SP, Brazil

**Keywords:** non-ionizing radiation, photosynthetic pigment, plant nutrition, plant growth stimulation, sustainable agriculture

## Abstract

Management practices that optimize physiological responses of crops can be applied in agriculture to achieve higher productivity in challenging environments limited by nutrient availability. Extremely Low Frequency (ELF), a type of non-ionizing radiation in the range of 0.3 to 300 Hz, interacts with biological systems and has potential applications in sustainable agriculture. This study evaluates the effects of ELF on morphophysiological parameters of soybean plants during the vegetative stage. Plants grown under controlled conditions were subjected to ELF treatments—Control, TA (which increases interatomic spacing), and TB (which decreases interatomic spacing)—in combination with three nutrient solution strengths (50%, 75%, and 100% of the Hoagland solution). Chlorophyll index, root and shoot length, and dry mass were measured at the end of experiment. ELF treatment significantly enhanced chlorophyll index, with treatment TB showing the greatest increase. This may suggest improved nutrient assimilation of key nutrients such as nitrogen and magnesium, which are critical for chlorophyll synthesis. These findings demonstrate the potential of ELF treatment to enhance plant physiological performance, even under nutrient-limited conditions. When combined with nutrient solutions, ELF exposure may promote plant health and growth by increasing chlorophyll index and may improve nutrient uptake. This approach represents a promising and sustainable strategy to boost crop productivity and resource use efficiency in agricultural systems.

## 1. Introduction

Extremely Low Frequencies (ELFs), defined within the range of 0.3 to 300 Hz, constitute a type of non-ionizing radiation [1]. Naturally present in the environment and considered safe, ELF are characterized by their ability to penetrate nearly all materials [2,3]. ELFs interact with biological systems by producing weak electric fields that alter cellular and molecular processes [4]. Although ELF do not carry enough energy to break chemical bonds, they can modulate the behavior of ions and charged particles, affecting biochemical reactions, including those involving radical pairs. ELF exposure can also alter local current density, electric field strength, and charge distribution on cell surfaces [5]. In biological molecules, ELF may induce resonance-like responses that potentially modify molecular functionality, such as through ion cyclotron resonance mechanisms. Additionally, ELF can influence the orientation and behavior of molecular dipoles, thereby affecting cellular signaling and metabolic processes [6].

Although the molecular targets of ELF in plants are still not fully established [7], available evidence supports membrane-associated processes and intracellular signaling as plausible early steps [5]. Experimental studies indicate that ELF-range magnetic fields can modulate membrane-associated electrical responses in leaves and influence plant physiological performance [8]. ELF exposure has also been associated with transient Ca^2+^ fluxes and changes in cytosolic Ca^2+^ levels, and Ca^2+^ signaling is a central regulator of transcriptional and physiological responses linked to nutrient acquisition and stress adaptation in plants [9]. In parallel, ELF-related changes in redox homeostasis have been discussed in relation to controlled reactive oxygen species (ROS) production, which functions as a signaling mechanism [9]. Although direct evidence for ELF-mediated regulation of specific transporters, aquaporins, or ion channels remains limited, these pathways are consistent with established plant signaling frameworks. In line with this context, an increasing number of studies report that ELF exposure can influence plant physiology, including growth and development, and can modify photosynthesis, water relations, and antioxidant metabolism [8,10,11,12].

Research by Souza-Torres et al. [10] showed that ELF-treated tomato seeds produced plants with improved water status, higher photosynthetic rates, and increased chlorophyll content compared to untreated controls. Similarly, Sudarti et al. [2] reported that ELF exposure led to greater leaf mass in tobacco plants, ultimately enhancing crop yield. In another study, ELF treatment of soybean under controlled conditions resulted in consistent improvements in seedling growth [13]. These promising findings position ELF as a potential strategy for sustainable and environmentally safe agriculture. With no reported harmful effects and no residual impact, ELF can complement existing conventional practices [3]. Optimizing crop physiological traits is critical for enhancing productivity while maintaining sustainability in modern agriculture [14]. Therefore, effective application of ELF to support crop nutrition could promote cleaner agricultural practices in line with global development and sustainability goals [15].

Considering its ability to enhance nutrient bioavailability, we hypothesize that ELF may modulate nutrient uptake dynamics and key morphophysiological traits, thereby improving nutrient use efficiency (NUE). This study assessed the effects of ELF treatment in combination with varying nutrient solution strengths on soybean seedlings during the vegetative stage.

## 2. Results

**Figure 1:**
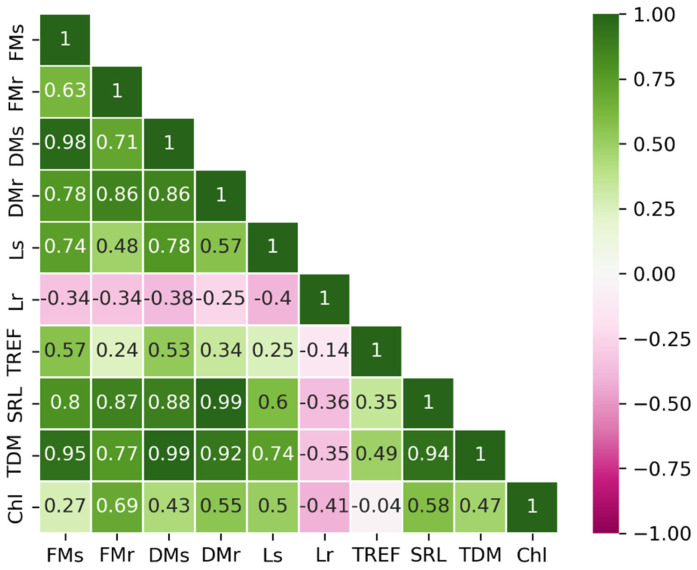
Pearson’s correlation matrix displaying the relationships between physiological parameters. FMs = fresh mass of the shoot; FMr = fresh mass of the root; DMs = dry mass of the shoot; DMr = dry mass of the root; Ls = shoot length; Lr = root length; TREF = number of trifoliate leaves; SRL = specific root length; TDM = total dry mass; Chl = chlorophyll.

**Figure 2:**
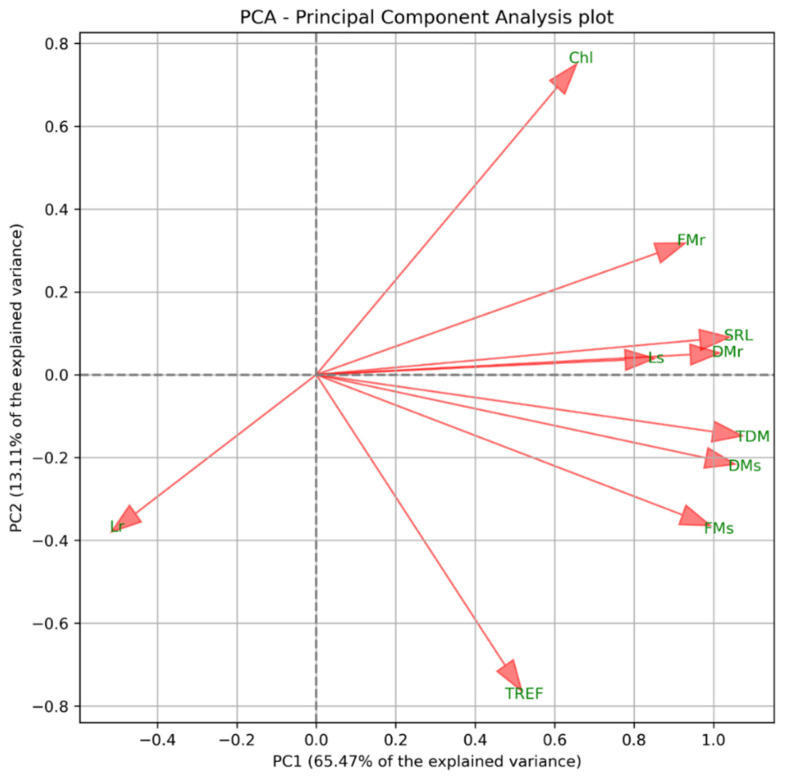
Principal Component Analysis (PCA) biplot showing the distribution of physiological parameters. FMs = fresh mass of the shoot; FMr = fresh mass of the root; DMs = dry mass of the shoot; DMr = dry mass of the root; Ls = shoot length; Lr = root length; TREF = number of trifoliate leaves; SRL = specific root length; TDM = total dry mass; Chl = chlorophyll.

After applying the interquartile range (IQR) filtering method, 30 observations were retained in the dataset. The correlation matrix of the ten variables is presented in Figure 1. A positive correlation was observed between FM, DM, SRL, and TDM, indicating that these variables are closely associated. In contrast, Lr exhibited a negative correlation with the remaining variables, implying that its increase may represent an independent or distinct trend. Meanwhile, Chl appeared to form a third group, showing no strong associations with other variables. These trends were consistent with the results of the PCA (Figure 2).

Based on the PCA loadings, the variables TDM, Lr, and Chl were selected for further analysis, due to their contribution to the main gradients of variation in the dataset. TDM exhibited the highest loading on PC1 (PC1 = 0.998), indicating that it contributes most strongly to explaining the primary axis of variation. Chl showed substantial loadings on both PC1 and PC2 (PC1 = 0.606; PC2 = 0.694), while Lr also influenced both components (PC1 = −0.455; PC2 = −0.335), suggesting its relevance to both the main and secondary trends.

TDM delineates the primary trend represented by PC1, Chl contributes substantially to both principal components, and Lr explains additional, distinct sources of variability. These variables, therefore, refine the interpretation of the bidimensional PCA projection presented in Figure 2, yielding a more comprehensive characterization of the dataset’s underlying structure.

**Figure 3:**
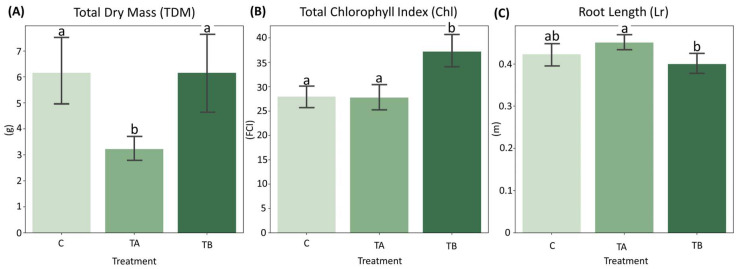
Barplot showing the distribution of: (**A**) Total Dry Mass (TDM), (**B**) Total Chlorophyll Index (Chl) and (**C**) Root Length (Lr) across treatments C, TA and TB. Different letters indicate statistically significant differences according to Tukey’s HSD test (*p* < 0.01). Treatments with the same lowercase letter do not differ significantly. n = 12 plants. FCI = Falker Chlorophyll Index.

Normality tests indicated that both Chl and Lr conformed to a normal distribution with homogeneous variances. In contrast, TDM violated the assumptions of normality and homogeneity (Supplementary Materials). Mean comparison analyses revealed that TDM did not differ significantly between control (C) and TB (which decreases interatomic spacing), whereas TA (which increases interatomic spacing) differed significantly from both treatments. For Chl, TB exhibited significant differences from both C and TA. Regarding Lr, no significant differences were detected between C and TB or between C and TA, although TA differed significantly from TB (Figure 3). Complete results of the mean comparison tests are presented in the Supplementary Materials.

The treatment TA exhibited the lowest TDM values (mean = 3.226 g), but did not differ significantly in Lr (mean = 0.451 m) from the Control (mean = 0.423 m) (Figure 3A,C). The observed reduction in shoot development, accompanied by a slight increase in root length, may reflect nutrient redistribution and adaptive strategies aimed at optimizing resource use under stress conditions. Conversely, TB exhibited an increase in Chl (mean = 37.212) and a lower Lr (mean = 0.400 m), contrasting with the trends observed in TA (Figure 3B,C)**.** The elevated Chl may indicate enhanced nutrient use efficiency (NUE), as chlorophyll is a key pigment in photosynthesis, and could reflect an adaptive response to improved nutrient uptake and utilization.

Additionally, the results across different nutrient solution concentrations did not show significant differences (Table 1), highlighting that even under more limiting soil conditions, TB was able to improve chlorophyll index.

## 3. Discussion

In the PCA, PC1 explains the variables contributing the greatest variance, representing the dominant trend in the dataset, while PC2, orthogonal to PC1, accounts for the second-highest variance. This framework facilitates the identification of underlying patterns and ensures that each component reflects a distinct source of variation [16,17].

The physiological responses observed under the different treatments highlight the contrasting adaptive strategies of soybean seedlings subjected to variable nutrient conditions and ELF exposure. In the TA treatment, plants exhibited reduced shoot biomass accompanied by a slight increase in root length, a trend commonly observed under suboptimal nutrient availability [18,19]. This growth allocation reflects a shift in the source–sink relationship, in which limited nutrient supply triggers preferential carbon partitioning toward root systems to enhance soil exploration and resource uptake [20,21]. Such responses are accentuated in confined environments, such as pot experiments, where restricted soil volume promotes nutrient redistribution from shoots to roots [22,23]. The stimulation of root elongation under these conditions can be considered an adaptive mechanism that improves nutrient foraging efficiency and may enhance tolerance to future abiotic stresses, including drought [24,25]. Therefore, inducing moderate nutrient limitation, as seen in TA, may contribute to modifications in root system architecture and be relevant for understanding plant responses under environmental stress conditions. However, this requires substantiation through mechanistic assessments, including detailed root system imaging (e.g., WinRHIZO-based root scans) and targeted hormone profiling (e.g., auxin quantification).

In contrast, the TB treatment promoted an increase in Chl, indicating higher leaf greenness under the conditions tested. As chlorophyll is a central pigment of the photosynthetic apparatus, this response is consistent with changes in plant nutritional status and photosynthetic potential and has been discussed as an indirect indicator of nutrient uptake and utilization [26].

Previous studies have demonstrated that ELF exposure, in association with optimized nutrient solutions, can enhance the metabolic utilization of nitrogen (N) and magnesium (Mg), two essential elements in chlorophyll biosynthesis [26,27]. The use of portable chlorophyll meters, such as the ClorofiLOG Falker^®^ CFL1030, has proven to be an effective method for assessing chlorophyll content and, indirectly, N status in plants [28,29,30]. Therefore, the elevated Chl observed in TB suggests an exploratory indication that this treatment may influence traits associated with plant nutritional status and may reflect physiological changes even in the absence of a direct increase in biomass production. However, confirming whether TB increases NUE through improved N/Mg uptake requires direct measurements, such as tissue elemental analysis as well as photosynthetic assays.

While higher Chl concentrations can enhance photosynthetic capacity by capturing more light energy [31], this does not necessarily translate into increased growth or yield, particularly during early vegetative [32,33]. The effect of ELF on chlorophyll synthesis and NUE likely represents a physiological adjustment aimed at optimizing resource use, which may become more pronounced under sustained or field-level conditions. Further research is warranted to evaluate whether the improvements in Chl observed under TB persist throughout later developmental stages and to investigate the kinetics of N and Mg uptake in relation to ELF exposure. In addition, targeted mechanistic studies are needed to evaluate whether ELF induces early membrane-associated signaling events, such as Ca^2+^ transients and reactive oxygen species (ROS) signatures, and whether subsequent responses involve adjustments in nutrient transport and assimilation, including nitrate and ammonium transporters (e.g., NRT1/NPF and AMT families) and key enzymes of nitrogen assimilation, such as glutamine synthetase/glutamate synthase (GS/GOGAT). In addition, potential effects on water transport regulation via aquaporins and on photosynthesis-related processes should be investigated.

Finally, the results suggest that ELF treatments can modulate plant physiological responses depending on nutrient availability: under nutrient limitation (TA), they can promote adaptive root development, while under enhanced nutrient conditions (TB), they can improve chlorophyll synthesis and nutrient assimilation. These findings align with previous studies reporting that ELF exposure can influence metabolic processes related to nutrient transport and photosynthesis [16,17], reinforcing its potential as a tool to optimize plant performance and sustainability in crop production systems.

## 4. Conclusions

The findings of this study indicate that ELF treatment, when combined with nutrient solutions, effectively increases chlorophyll index in soybean seedlings. These results also demonstrate that ELF treatments can be applied in soybean cultivation as a management practice to support plant development and productivity, particularly under nutrient-limited conditions. The increase in chlorophyll levels can be linked to the enhanced nutrient use efficiency and photosynthetic activity, supporting the hypothesis that ELF can optimize vegetative growth and plant performance, offering a promising tool for sustainable strategy to improve crop performance in the field. However, further studies, including transcriptomic approaches and the mechanistic analyses outlined above, are required to elucidate the underlying molecular mechanisms and regulatory pathways involved in ELF-mediated responses.

## 5. Materials and Methods

### 5.1. Experimental Design

The experiment was conducted using a completely randomized factorial design (3 × 3 × 4) with four replicates per treatment, totaling 36 soybean plants. Treatments consisted of combinations of three ELF exposures—Control, TA (which increases interatomic spacing), and TB (which decreases interatomic spacing)—and three nutrient solution strengths (50%, 75%, and 100% of a modified Hoagland and Arnon solution). The composition of the nutrient solutions, including the sources of each element, is detailed in Table 2. The nine resulting treatment combinations were TA + 50%, TA + 75%, TA + 100%, TB + 50%, TB + 75%, TB + 100%, Control + 50%, Control + 75%, and Control + 100%.

### 5.2. Growth Condition

The experiment was conducted across three growth rooms under controlled edaphoclimatic conditions suitable for soybean cultivation. GreenPower LED toplighting linear lamps (Philips, Eindhoven, The Netherlands) provided light in the 400–700 nm range, with the photosynthetic photon flux density (PPFD) measured at canopy height using a quantum sensor (LI-COR Biosciences, Lincoln, NE, USA), yielding approximately 700 µmol·m^−2^·s^−1^ and a 14 h photoperiod. Daytime and nighttime temperatures were maintained at approximately 27 °C and 17 °C, respectively, using an air conditioning system to ensure stable growth conditions.

Irrigation was performed daily with a modified Hoagland and Arnon [34] nutrient solution, with the pH adjusted to 5.9, applied according to the substrate’s field capacity to ensure percolation from the bottom of the pots (Table 2). Each plant was grown in a 3.0 L pot filled with an inert substrate (vermiculite) to maintain consistent nutrient availability. Conventional soybean (*Glycine max*), cultivar BRS511-EMBRAPA, was used and grown for 45 days, until the end of the vegetative stage.

### 5.3. ELF Treatment

**Figure 4:**
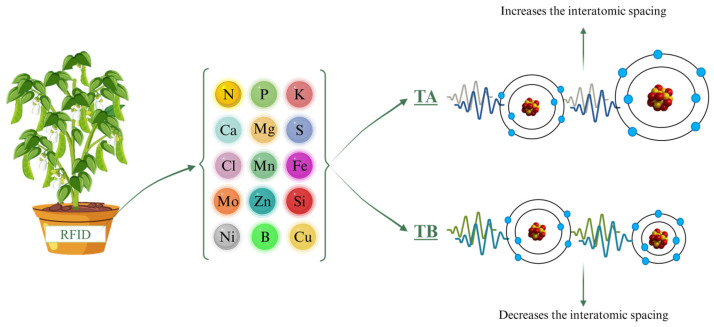
Experimental schematic illustrating soybean plants exposed to ELF treatments—Control, TA (which increases interatomic spacing), and TB (which decreases interatomic spacing)—across different nutrient solution strengths. Frequency treatments depict how ELF modifies interatomic spacing, potentially influencing nutrient bioavailability and uptake. RFID = Radio-Frequency Identification (Effatha Agro, São Paulo, Brazil).

Throughout the experiment, ELF treatments were applied to each plant pot using a contact RFID (Radio-Frequency Identification) card (ISO 144333 A, Effatha, Santo André, SP, Brazil). The ELF technology used in this study operates at 0–100 Hz and generates a magnetic flux density of 45 µT, and it is intended to influence molecular-scale interactions [35,36]. Using an algorithm informed by periodic table data, the system generates specific frequency sequences that either increase or decrease interatomic spacing, thereby influencing molecular structures. Each molecule, with its unique atomic arrangement, requires a tailored sequence of frequencies, and the timing of application is critical to achieve the intended effects. The sequences were customized for each nutrient treatment, targeting the specific cations and anions needed by the plants (Figure 4). Nutrient solutions were provided at 50%, 75%, or 100% of the standard Hoagland solution (Figure 4). The algorithmic parameters of the system are proprietary and cannot be disclosed, consistent with best practices for ensuring transparency and reproducibility within the limits of intellectual property protections [35].

Plant pots were distributed across three separate growth rooms to prevent interference between treatments through proximity or balcony contact. The manufacturer specified the interference radius of the RFID cards used in this study, ensuring adequate separation and avoiding cross-contamination of treatments.

### 5.4. Plant Analysis

#### 5.4.1. Trefoil Number (TREF)

Trefoil number was recorded prior to harvest to assess the phenological stage of the plants. Only fully expanded trifoliate leaves, starting from the base of the plant and progressing upward, were identified and counted [37].

#### 5.4.2. Chlorophyll (Chl) Index

Total chlorophyll index was measured in triplicate for each plant using a portable chlorophyll meter (ClorofiLOG FL1030, Falker^®^, Porto Alegre, Brazil), allowing non-destructive readings, and expressed as Falker Chlorophyll Index (FCI) units. The instrument was auto-calibrated prior to readings using its internal dark/clear reference. To capture an accurate representation of Chl distribution, leaves were sampled from three distinct regions of the plant—base, middle, and top—covering both older basal leaves and younger apical leaves. This approach aimed to account for potential variations in nutrient allocation and photosynthetic activity across the plant [38].

#### 5.4.3. Root (Lr) and Shoot (Ls) Length

After harvest, plants were carefully removed from the pots, and the growth medium was gently washed from the roots to minimize damage. Root and shoot lengths were measured using a ruler. Shoot length was recorded from the base of the stem (soil level) to the tip of the tallest point of the plant. The root system was laid flat, and root length was measured from the root–shoot junction (stem base) to the tip of the longest root [39].

#### 5.4.4. Fresh (FM) and Dry Mass (DM)

Immediately after harvesting, the fresh mass of the shoot and root systems was determined. Each plant part was weighed separately using an analytical balance to ensure accuracy. To assess plant growth, total dry mass was measured by placing the separated shoot and root parts in paper bags and drying them in an oven at 70 °C for 72 h. Dry mass was recorded using an analytical balance, and total dry mass (TDM) was calculated as the sum of the shoot and root dry mass [40].

#### 5.4.5. Specific Root Length (SRL)

Specific Root Length (SRL) was calculated to relate Root Length to Root Dry Mass, providing an estimate of root biomass efficiency. SRL was determined following the method of Ostonen et al. [41], using the formula:

$$SRL=\;\frac{Root\;Lenght\;(cm)}{Root\;Dry\;Mass\;(g)}$$

### 5.5. Statistical Analysis

Statistical analyses were performed using Python (version 8.10.0) in Jupyter Notebook (version 6.5.2) [42]. Data were initially screened to remove potential outliers. Pearson’s correlation coefficient (r) was calculated, and principal component analysis (PCA) was conducted to reduce dimensionality and identify variables contributing most to dataset variance. Based on both the correlation results and PCA loadings of the first two principal components, three variables were selected for further analysis.

Normality of data was assessed using the Shapiro–Wilk test, and homogeneity of variances was evaluated with Levene’s test to determine the appropriate subsequent analyses. Parametric data were analyzed using analysis of variance (ANOVA), followed by Tukey’s Honest Significant Difference (HSD) test for multiple comparisons. For non-parametric data, Kruskal–Wallis tests were applied, followed by Dunn’s post hoc test.

## Figures and Tables

**Table 1:** Tukey’s post hoc analysis of chlorophyll among nutrient solution concentrations (50%, 75%, and 100%).

Multiple Comparison of Means–Chl (Dilutions)-Tukey Hsd, Fwer = 0.05						
Group1	Group2	Meandiff	*p*-adj	Lower	Upper	Reject
50	75	1.6417	0.4218	−1.5593	4.8426	False
50	100	0.1386	0.9932	−2.9535	3.2313	False
75	100	−1.5028	0.5215	−4.9104	1.9049	False

Chl = chlorophyll; Meandiff = mean differences; *p*-adj = *p*-value. Significant differences (*p* < 0.05).

**Table 2:** Nutrient sources and concentration (g L^−1^) used in the preparation of the nutrient solution.

Nutrient	50%	75%	100%
Ca(NO_3_)_2_·4H_2_O	0.47	0.705	0.94
NH_4_H_2_PO_4_	0.115	0.1725	0.23
KNO_3_	0.305	0.4575	0.61
MgSO_4_·7H_2_O	0.245	0.3675	0.49
Fe-EDTA	0.0125	0.0188	0.025
H_3_BO_3_	0.0016	0.0023	0.0031
MnCl_2_·4H_2_O	0.001	0.0015	0.002
ZnSO_4_·7H_2_O	0.0001	0.0002	0.0002
CuSO_4_·5H_2_O	0.00005	0.00008	0.0001
Na_2_MoO_4_·2H_2_O	0.00005	0.00008	0.0001
NaCl	0.0029	0.0044	0.0058

## Data Availability

Data are contained within the article and Supplementary Materials.

## Supplementary Materials

The following supporting information can be downloaded at: https://www.mdpi.com/article/10.3390/plants15030495/s1.

## Abbreviations

ELF, Extremely Low Frequency; GS, Glutamine Synthetase; GOGAT, Glutamate Synthase; ROS, Reactive Oxygen Species; NUE, Nutrient Use Efficiency; TREF, Trefoil Number; Chl, Chlorophyll; Lr, Root Length; Ls, Shoot Length; FM, Fresh Mass (DM); DM, Dry Mass; SRL, Specific Root Length; TDM, Total Dry Mass; PCA, Principal Component Analysis; HSD, Tukey’s Honest Significant Difference.
