# Supplementary material for: Extremely Low Frequency Radiation Enhances Soybean Chlorophyll Index and Nutrient Use Efficiency Under Suboptimal Conditions
Source: Plants (Basel). 2026 Feb 5;15(3):495. doi: 10.3390/plants15030495 (PMC12899631; doi:10.3390/plants15030495)
Supplement: Supplementary file 1 [file plants-15-00495-s001.zip › plants-4051213-supplementary.pdf]

Supplementary Figures

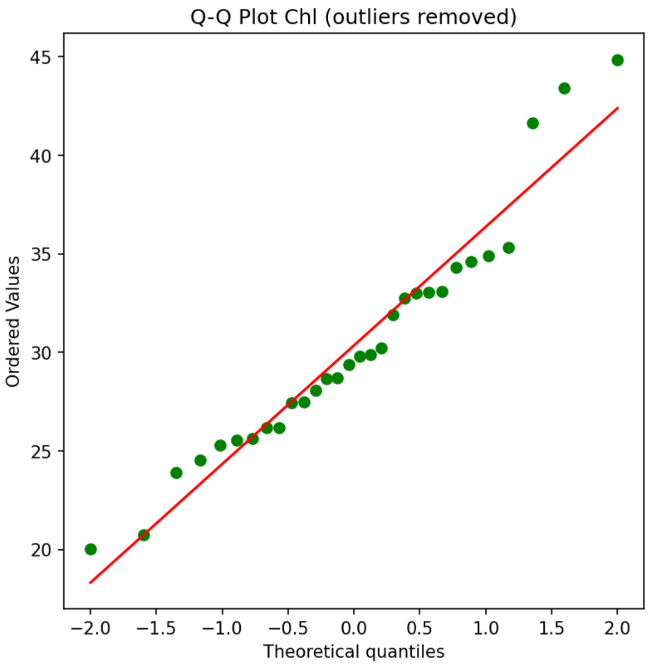

Supplementary Figure S1. Q–Q plot of chlorophyll content (Chl) after outlier removal. The plot illustrates the normality of the chlorophyll content distribution across treatments. The close alignment of data points along the red reference line indicates that the data follow a normal distribution, confirming the homogeneity of variance for Chl.

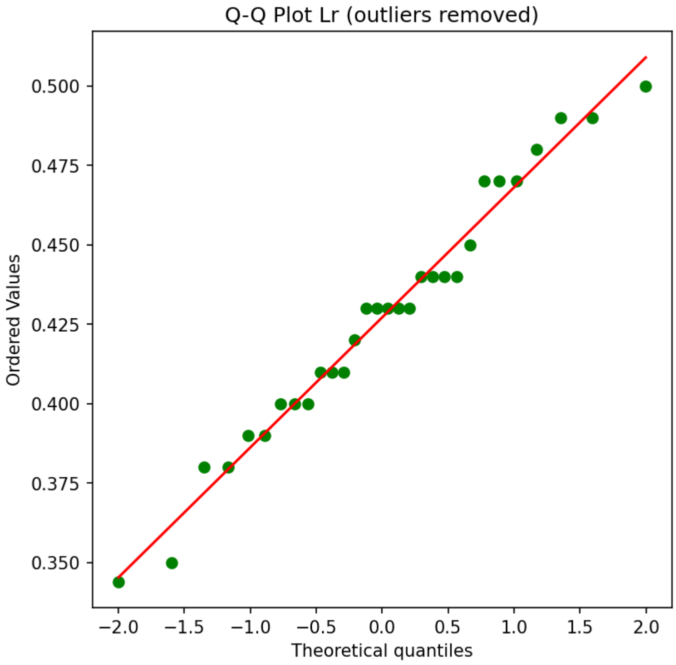

Supplementary Figure S2. Q–Q plot of root length (Lr) after outlier removal. The plot illustrates the normality of the root length data, with data points closely following the red reference line, indicating a normal distribution and consistent variance among Lr measurements.

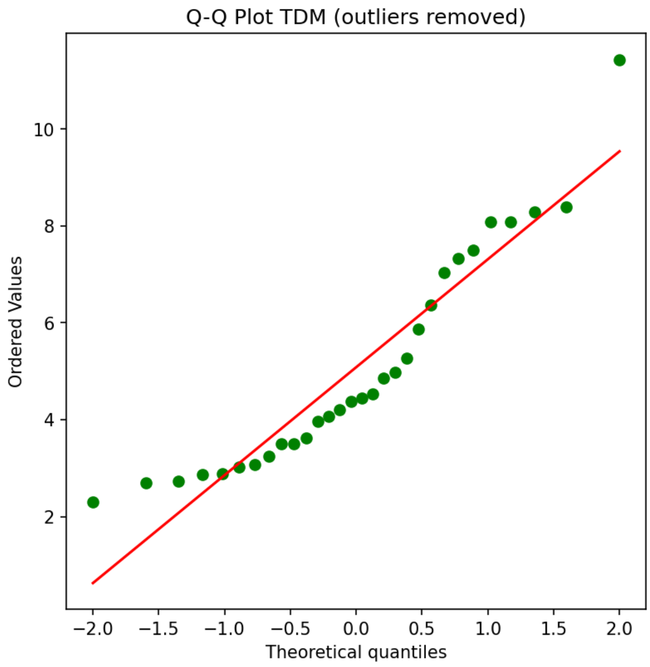

Supplementary Figure S3. Q–Q plot of total dry mass (TDM) after outlier removal, showing deviations from the red line at upper quantiles, which suggest non-normality and heterogeneity of variance.

Supplementary Tables

Supplementary Table S1. Analysis of variance (ANOVA) for fresh mass of the shoot (FMs) as affected by treatment, nutrient solution, and their interaction (treatment × nutrient solution).

ANOVA FMs, Fwer=0.05				
	**sum sq**	**df**	**F**	**PR(>F)**
T	1015.671138	2.0	15.943509	0.000061
S	304.834433	2.0	4.785142	0.019394
T:S	195.314847	4.0	1.532979	4.4063
Residues	668.895833	21.0	NaN	NaN

FMs = Fresh mass of the shoot; T = Treatment; S = Nutrient Solution; sum sq = sum of squares; df = degrees of freedom; F = F-value; PR(>F) = p-value. Significant differences were considered at $p < 0.05$.

Supplementary Table S2. Analysis of variance (ANOVA) for fresh mass of the root (FMr) as affected by treatment, nutrient solution, and their interaction (treatment × nutrient solution).

ANOVA FMr, Fwer=0.05				
	**sum sq**	**df**	**F**	**PR(>F)**
T	1042.951698	2.0	7.646909	0.003199
S	740.601341	2.0	5.430080	0.012566
T:S	252.778245	4.0	0.926683	0.467273
Residues	1432.081000	21.0	NaN	NaN

FMr = Fresh mass of the root; T = Treatment; S = Nutrient Solution; sum sq = sum of squares df = degrees of freedom; F = F-value; PR(>F) = p-value. Significant differences were considered at $p < 0.05$.

Supplementary Table S3. Analysis of variance (ANOVA) for dry mass of the shoot (DMs) as affected by treatment, nutrient solution, and their interaction (treatment × nutrient solution).

ANOVA DMs, Fwer=0.05				
	**sum sq**	**df**	**F**	**PR(>F)**
T	33.167595	2.0	14.186128	0.000126
S	15.215611	2.0	6.507876	0.006319
T:S	8.677942	4.0	1.855823	0.155904
Residues	24.549317	21.0	NaN	NaN

DMs = Dry mass of the shoot; T = Treatment; S = Nutrient Solution; sum sq = sum of squares df = degrees of freedom; F = F-value; PR(>F) = p-value. Significant differences were considered at $p < 0.05$.

Supplementary Table S4. Analysis of variance (ANOVA) for dry mass of the root (DMr) as affected by treatment, nutrient solution, and their interaction (treatment × nutrient solution).

ANOVA DMr, Fwer=0.05				
	**sum sq**	**df**	**F**	**PR(>F)**
T	2.953250	2.0	6.800618	0.005283
S	2.523474	2.0	5.810949	0.009805
T:S	1.069345	4.0	1.231222	0.327810
Residues	4.559750	21.0	NaN	NaN

DMr = Dry mass of the root; T = Treatment; S = Nutrient Solution; sum sq = sum of squares df = degrees of freedom; F = F-value; PR(>F) = p-value. Significant differences were considered at $p < 0.05$.

Supplementary Table S5. Analysis of variance (ANOVA) for total dry mass (TDM) as affected by treatment, nutrient solution, and their interaction (treatment × nutrient solution).

ANOVA TDM, Fwer=0.05				
	**sum sq**	**df**	**F**	**PR(>F)**
T	55.600677	2.0	13.136087	0.000200
S	29.940669	2.0	7.073713	0.004481
T:S	14.172004	4.0	1.674122	0.193503
Residues	44.443000	21.0	NaN	NaN

TDM = Total dry mass; T = Treatment; S = Nutrient Solution; sum sq = sum of squares df = degrees of freedom; F = F-value; PR(>F) = p-value. Significant differences were considered at $p < 0.05$.

Supplementary Table S6. Analysis of variance (ANOVA) for length of the shoot (Ls) as affected by treatment, nutrient solution, and their interaction (treatment × nutrient solution).

ANOVA Ls, Fwer=0.05				
	**sum sq**	**df**	**F**	**PR(>F)**

T	0.025299	2.0	6.506053	0.006326
S	0.006298	2.0	1.619680	0.221733
T:S	0.010286	4.0	1.322557	0.294185
Residues	0.040829	21.0	NaN	NaN

Ls = Length of the shoot; T = Treatment; S = Nutrient Solution; sum sq = sum of squares df = degrees of freedom; F = F-value; PR(>F) = p-value. Significant differences were considered at $p < 0.05$.

Supplementary Table S7. Analysis of variance (ANOVA) for length of the root (Lr) as affected by treatment, nutrient solution, and their interaction (treatment × nutrient solution).

ANOVA Lr, Fwer=0.05				
	**sum sq**	**df**	**F**	**PR(>F)**
T	0.011246	2.0	4.903660	0.017883
S	0.002736	2.0	1.193044	0.323034
T:S	0.006845	4.0	1.492436	0.240314
Residues	0.024080	21.0	NaN	NaN

Lr = Length of the root; T = Treatment; S = Nutrient Solution; sum sq = sum of squares df = degrees of freedom; F = F-value; PR(>F) = p-value. Significant differences were considered at $p < 0.05$.

Supplementary Table S8. Analysis of variance (ANOVA) for specific root length (SRL) as affected by treatment, nutrient solution, and their interaction (treatment × nutrient solution).

ANOVA SRL, Fwer=0.05				
	**sum sq**	**df**	**F**	**PR(>F)**
T	20.507659	2.0	8.540960	0.001931
S	15.999532	2.0	6.663430	0.005743
T:S	5.667548	4.0	1.180201	0.348148
Residues	25.211501	21.0	NaN	NaN

SRL = Specific root length; T = Treatment; S = Nutrient Solution; sum sq = sum of squares df = degrees of freedom; F = F-value; PR(>F) = p-value. Significant differences were considered at $p < 0.05$.

Supplementary Table S9. Analysis of variance (ANOVA) for number of trefoils (TREF) as affected by treatment, nutrient solution, and their interaction (treatment × nutrient solution).

ANOVA TREF, Fwer=0.05				
	sum sq	df	F	PR(>F)
T	1.602922	2.0	0.554858	0.582344
S	5.246609	2.0	1.816134	0.187286
T:S	0.931421	4.0	0.161208	0.955621
Residues	30.333333	21.0	NaN	NaN

TREF = Number of trefoils; T = Treatment; S = Nutrient Solution; sum sq = sum of squares df = degrees of freedom; F = F-value; PR(>F) = p-value. Significant differences were considered at $p < 0.05$.

Supplementary Table S10. Analysis of variance (ANOVA) for chlorophyll index (Chl) as affected by treatment, nutrient solution, and their interaction (treatment × nutrient solution).

ANOVA Chl, Fwer=0.05				
	sum sq	df	F	PR(>F)
T	432.408527	2.0	19.047679	0.000019
S	211.729853	2.0	9.326741	0.001263
T:S	64.670817	4.0	1.424381	0.260622
Residues	238.364444	21.0	NaN	NaN

Chl = Chlorophyll index; T = Treatment; S = Nutrient Solution; sum sq = sum of squares df = degrees of freedom; F = F-value; PR(>F) = p-value. Significant differences were considered at $p < 0.05$.

Supplementary Table S11. Tukey's post-hoc analysis for chlorophyll content (Chl) among different treatments.

Multiple Comparison of Means<br>Chl - Tukey Hsd, Fwer=0.05						
Group1	Group2	Meandiff	p-adj	Lower	Upper	Reject
C	TA	-0.1788	0.9949	-4.7951	4.4375	False
C	TB	9.258	0.0003	4.2275	14.2884	True
TA	TB	9.4367	0.0002	4.4063	14.4672	True

C = Control; TA = Treatment A; TB = Treatment B; Chl = chlorophyll index; Meandiff = mean differences; p-adj = p-value. Significant differences were considered at $p < 0.05$.

Supplementary Table S12. Tukey's post-hoc analysis for Root Length (Lr) among different treatments.

**Multiple Comparison of Means**						
**Lr - Tukey Hsd, Fwer=0.05**						
**Group1**	**Group2**	**Meandiff**	**p-adj**	**Lower**	**Upper**	**Reject**
C	TA	-0.0278	0.1736	-0.0095	0.0651	False
C	TB	-0.0231	0.3513	-0.0638	0.0176	False
TA	TB	-0.0509	0.012	-0.0916	-0.0102	True

C = Control; TA = Treatment A; TB = Treatment B. Lr = root lenght; Meandiff = mean differences; p-adj = p-value. Significant differences were considered at $p < 0.05$.

Supplementary Table S13. Kruskal-Wallis and Dunn test with Bonferroni adjustment for Total Dry Mass (TDM) among different treatments.

**Multiple Comparison of Means**			
**TDM - Kruskal-Wallis And Dunn Test (Bonferroni adjustment)**			
	C	TA	TB
C	1.000000	0.001599	1.000000
TA	0.001599	1.000000	0.003388
TB	1.000000	0.003388	1.000000

C = Control; TA = Treatment A; TB = Treatment B. Significant differences were considered at $p < 0.05$.
